# Indocyanine Green Angiography for Objective Perfusion Monitoring After Hyaluronic Acid Filler‐Induced Facial Vascular Occlusion

**DOI:** 10.1111/jocd.71097

**Published:** 2026-08-03

**Authors:** Guangdi Li, Guiwen Zhou, Miao Wang, Hongfan Ding, Chongli Yu, Qiang Fu, Minliang Chen

**Affiliations:** ^1^ Plastic and Reconstructive Surgery Department The Fourth Medical Center of Chinese PLA General Hospital Beijing China; ^2^ Trauma Repair and Tissue Regeneration Center, Plastic Surgery Hospital Chinese Academy of Medical Sciences, Peking Union Medical College Beijing China; ^3^ Department of Plastic and Reconstructive Surgery, Plastic Surgery Hospital Chinese Academy of Medical Sciences, Peking Union Medical College Beijing China; ^4^ School of Medicine Nankai University Tianjin China

**Keywords:** cutaneous ischemia, hyaluronic acid filler, hyaluronidase, indocyanine green angiography, perfusion monitoring, vascular occlusion

## Abstract

**Background:**

Hyaluronic acid (HA) filler‐induced facial vascular occlusion may cause cutaneous ischemia, necrosis, and disfigurement. Clinical assessment and imaging‐guided hyaluronidase‐based intervention remain central, but vessel‐level localization does not directly quantify downstream tissue perfusion after rescue treatment.

**Objectives:**

To evaluate indocyanine green angiography (ICGA) as an adjunctive tissue‐level tool for monitoring perfusion changes after initial rescue treatment.

**Materials and Methods:**

This exploratory single‐center retrospective feasibility study included patients with clinically diagnosed HA filler‐induced facial vascular occlusion who underwent paired ICGA before and after initial rescue treatment between July 2025 and March 2026. ICGA‐derived parameters included ingress rate, time to peak (TPP), and curve ingress. Curve ingress percent change was calculated as the change in curve ingress divided by baseline curve ingress.

**Results:**

Twenty‐six patients were included. After initial rescue treatment, median ingress rate increased from 12.50 to 16.20, with a median change of 4.85 (IQR, 3.00–8.57; *p* < 0.001). Median TPP shortened from 31.46 to 23.87 s, with a median change of −6.09 s (IQR, −11.69 to −3.93; *p* < 0.001). Median curve ingress increased from 63515.95 to 77862.55, with a median change of 14432.45 (IQR, 9891.28–20269.65; *p* < 0.001). The two patients requiring treatment escalation showed the lowest percent changes in curve ingress.

**Conclusions:**

ICGA demonstrated early tissue‐level perfusion changes after initial rescue treatment. Limited early curve ingress improvement may support closer surveillance or timely escalation when interpreted with clinical findings.

**Trial Registration:**

ClinicalTrials.gov, NCT07581652

## 1 Introduction

The widespread use of hyaluronic acid (HA) fillers has been accompanied by increasing attention to rare but potentially devastating vascular complications. Among these, HA filler‐induced facial vascular occlusion is among the most serious adverse events because delayed recognition or insufficient rescue may result in cutaneous ischemia, tissue necrosis, disfigurement, and the need for subsequent wound or reconstructive management [1, 2]. Although uncommon, the functional and aesthetic consequences of this complication can be substantial, making timely recognition and prompt intervention essential.

In current clinical practice, the diagnosis and early management of filler‐induced vascular occlusion rely primarily on clinical findings, including acute pain, blanching, livedoid discoloration, delayed capillary refill, skin temperature change, and evolving ischemic skin changes [3, 4, 5]. These signs remain central to bedside decision‐making and immediate treatment. However, clinical assessment is partly subjective and may change dynamically during the early post‐treatment period. As a result, it can be difficult to determine the extent of cutaneous perfusion compromise objectively or to evaluate whether initial rescue treatment has restored tissue perfusion adequately.

Imaging‐based assessment may provide complementary information in selected cases. Ultrasound and color Doppler imaging are useful for identifying vascular anatomy, detecting flow abnormalities, and guiding targeted hyaluronidase delivery [6, 7]. However, vessel‐level localization does not necessarily provide direct information regarding downstream cutaneous perfusion within the affected tissue territory. This distinction is clinically important because successful vessel localization and targeted drug delivery do not always confirm adequate restoration of skin perfusion. Therefore, an adjunctive method that visualizes tissue‐level perfusion before and after rescue treatment may be valuable for objective treatment‐response assessment, particularly when clinical signs are equivocal or when the adequacy of reperfusion is uncertain.

Indocyanine green angiography (ICGA) is a well‐established technique for real‐time visualization and quantitative assessment of tissue perfusion [8, 9, 10]. In reconstructive and plastic surgery, ICGA has been widely applied for perforator localization, flap design optimization, intraoperative perfusion assessment, and evaluation of tissue viability. Previous studies have shown that ICGA can provide dynamic and quantifiable vascular information and may outperform conventional handheld Doppler for perforator identification, while post‐processing tools such as SPY‐Q allow objective analysis of perfusion intensity and perfusion territories [11]. In facial reconstructive surgery, ICGA has also been used to identify superficial temporal vessels, guide flap design, and assess distal flap perfusion in head and neck reconstruction [12, 13]. These applications suggest that ICGA may be suitable for monitoring superficial cutaneous and subcutaneous perfusion in facial vascular compromise.

Despite these potential advantages, the role of ICGA in HA filler‐induced facial vascular occlusion remains insufficiently defined. In this setting, the potential value of ICGA lies in providing objective, dynamic, and quantitative information regarding downstream tissue reperfusion after rescue intervention. However, evidence remains limited regarding how ICGA‐derived parameters change immediately after treatment and whether insufficient early improvement in tissue‐level perfusion may identify patients requiring closer surveillance or treatment escalation.

Accordingly, this study aimed to evaluate the feasibility of ICGA and explore its potential clinical relevance for objective perfusion monitoring in patients with HA filler‐induced facial vascular occlusion. Specifically, we characterized paired changes in ICGA‐derived perfusion parameters before and after initial rescue treatment, including ingress rate, TPP, and curve ingress. We also explored whether limited early curve ingress improvement corresponded to clinical evidence of inadequate reperfusion and subsequent treatment escalation.

## 2 Materials and Methods

### 2.1 Study Design and Patient Selection

This exploratory single‐center retrospective feasibility study was designed to evaluate paired ICGA‐derived tissue‐level perfusion changes before and after initial rescue treatment in patients with clinically diagnosed HA filler‐induced facial vascular occlusion. Patients who presented to our institution between July 2025 and March 2026 were eligible for review if they had compatible clinical manifestations of acute vascular compromise and underwent paired ICGA assessment before and after initial rescue treatment. The diagnosis was established clinically based on a recent history of HA filler injection together with acute findings suggestive of vascular compromise, including pain, blanching, livedoid discoloration, delayed capillary refill, skin temperature change, and/or evolving ischemic skin changes.

**FIGURE 1:**
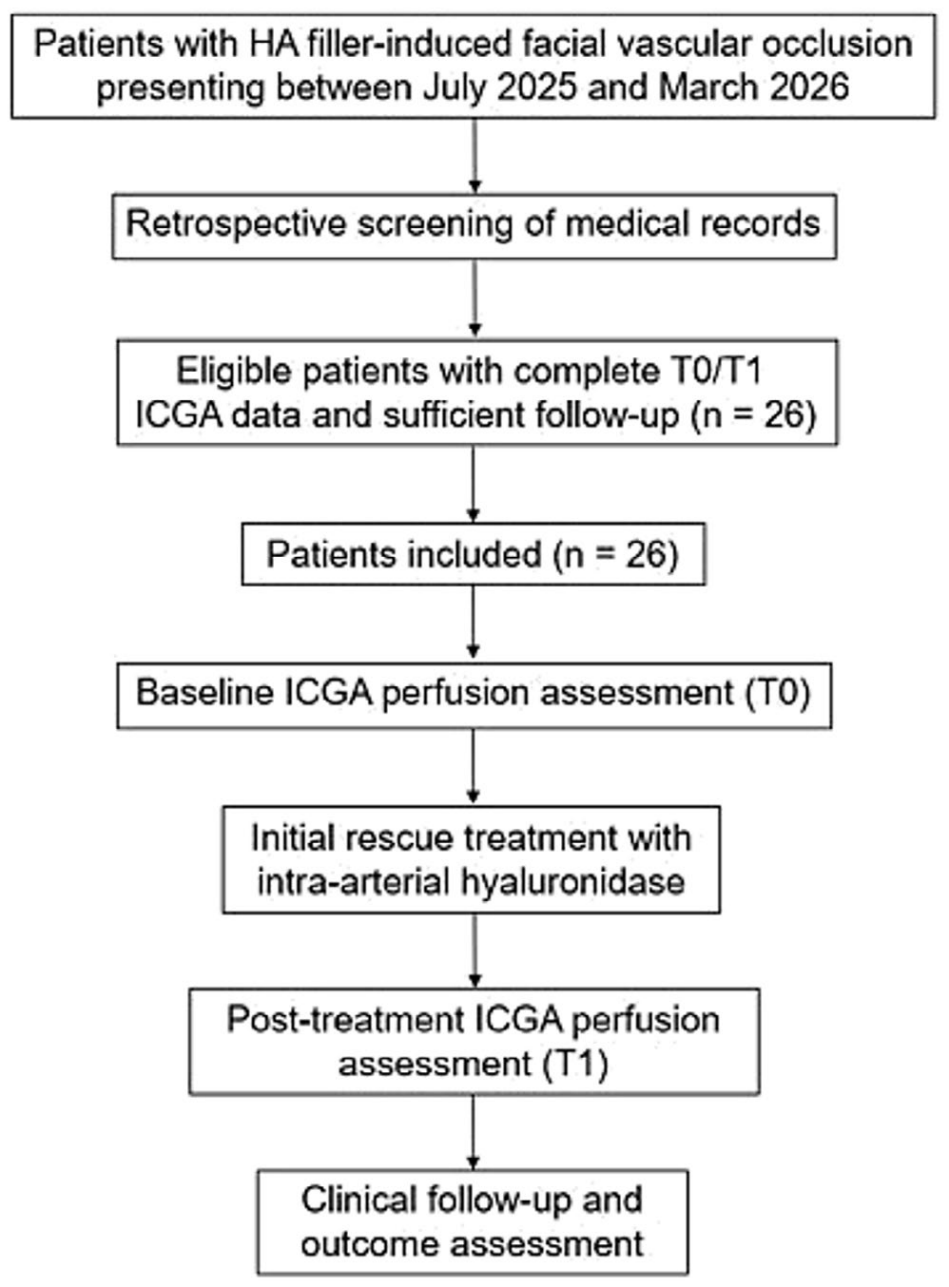
Study flow diagram. Flow diagram showing patient screening, reasons for exclusion, paired ICGA assessment, initial rescue treatment, and follow‐up. HA, hyaluronic acid; ICGA, indocyanine green angiography; T0, baseline assessment; T1, post‐treatment assessment.

Eligible cases were identified through a retrospective review of institutional medical records. Of 54 patients assessed for eligibility during the study period, 28 were excluded, including 21 whose vascular occlusion was not attributable to HA filler injection, six with incomplete paired ICGA data, and 1 with insufficient follow‐up documentation. The final analysis included 26 patients with clinically diagnosed HA filler‐induced facial vascular occlusion, complete paired ICGA data before and after initial rescue treatment, and sufficient follow‐up information for clinical outcome assessment. The study flow is shown in Figure 1.

The study protocol was approved by the Ethics Committee of Plastic Surgery Hospital, Chinese Academy of Medical Sciences (approval no. 2026042018584440) and was conducted in accordance with the Declaration of Helsinki. This study was registered at ClinicalTrials.gov (identifier: NCT07581652). Written informed consent was obtained for the publication of identifiable clinical and imaging materials when applicable.

### 2.2 Clinical Management and ICGA

Initial rescue management was initiated on the basis of clinical diagnosis of vascular compromise and was not delayed for imaging. ICGA was used as an adjunctive perfusion‐monitoring tool to assess the affected cutaneous territory and the early reperfusion response. ICGA was performed at two predefined peri‐treatment time points in the operating room. Baseline ICGA was obtained before intra‐arterial hyaluronidase injection (T0) when this could be performed without delaying urgent management. Post‐treatment ICGA was generally obtained approximately 15 min after completion of the initial intra‐arterial hyaluronidase injection (T1) to assess early downstream perfusion changes. Exact minute‐level T1 intervals were not consistently recorded in the retrospective medical records.

To minimize acquisition‐related variation between paired T0 and T1 ICGA assessments, a standardized workflow was used. Patient position, imaging angle, camera working distance, field of view, SPY system settings, room environment, and ambient lighting were kept as consistent as feasible within each patient; room lights were turned off during image acquisition. The indocyanine green dose, injection method, injection speed, and saline flush protocol were also kept consistent between paired acquisitions. Quantitative analyses were performed using the same SPY‐Q software workflow for paired images. Because no post hoc normalization to absolute maximum or minimum fluorescence intensity was applied, ICGA‐derived intensity parameters were interpreted primarily as paired within‐patient changes rather than as absolute cross‐patient perfusion values.

Initial rescue treatment consisted of percutaneous intra‐arterial hyaluronidase injection according to the suspected vascular territory and our previously reported institutional protocol. The hyaluronidase product used was Hyaluronidase for Injection (Shanghai First, 1500 IU/vial; Shanghai Shangyao No. 1 Biochemical Pharmaceutical Co. Ltd., Shanghai, China). All included patients received a uniform initial intra‐arterial hyaluronidase dose of 1500 IU. This approach was used as a selected institutional rescue strategy in referred patients with persistent clinical evidence of vascular compromise.

Most patients were referred after initial management at outside institutions, and many had already received local hyaluronidase treatment before presentation but continued to show persistent ischemic symptoms and signs. In this context, intra‐arterial delivery was considered by the treating team as a targeted rescue option for the suspected vascular territory; however, conventional early local hyaluronidase‐based treatment remains central to management and should not be delayed. When used, ultrasound or color Doppler imaging served as a procedural guidance tool for vascular localization and targeted arterial puncture, rather than as a standardized comparator modality for ICGA. The target artery was selected according to the clinically involved region and available imaging‐based localization, including the facial artery or supratrochlear artery [14, 15]. No standardized ultrasound‐derived quantitative parameters, such as vessel diameter, flow velocity, waveform pattern, resistive index, embolus length, or residual flow grading, were prespecified or consistently recorded in this retrospective cohort. Intraluminal placement was confirmed by positive blood aspiration before slow intra‐arterial injection of hyaluronidase. Adjunctive pharmacologic and wound‐supportive treatments were administered according to clinical presentation and institutional practice.

### 2.3 ICGA Acquisition and Quantitative Perfusion Parameters

ICGA was performed using the SPY Elite Imaging System (Novadaq Technologies Inc., Richmond, BC, Canada). A weight‐based dose of indocyanine green was administered intravenously and flushed with saline before video recording of perfusion in the target area. Quantitative analysis was performed using the SPY‐Q analysis toolkit [16, 17].

The ICGA‐derived perfusion parameters included ingress rate, TPP, and curve ingress. Ingress rate represents the maximum slope of fluorescence intensity increase and was reported in units/s. TPP represents the interval from initial fluorescence appearance to peak fluorescence intensity and was reported in seconds. Curve ingress represents the area under the fluorescence‐time curve from fluorescence onset to peak intensity and was reported in units·s. For each parameter, the absolute change between T1 and T0 was calculated as the post‐treatment value minus the baseline value. Curve ingress percent change was calculated as [(curve ingress at T1 − curve ingress at T0)/curve ingress at T0] × 100%.

Regions of interest were selected within the clinically affected cutaneous territory based on ICGA images and corresponding clinical photographs. When serial images were available, the same or anatomically comparable region was used for baseline and post‐treatment measurements to improve consistency. ROI area was not specifically measured or consistently recorded in the retrospective SPY‐Q outputs; therefore, curve ingress was interpreted primarily as a within‐patient paired parameter rather than as an absolute cross‐patient or per‐unit‐area perfusion metric.

### 2.4 Data Collection

Clinical data were extracted from medical records, including age, sex, body mass index (BMI), affected facial region, time from filler injection to initial rescue treatment, prior hyaluronidase treatment before presentation, treatment details, treatment escalation, final clinical outcome, and treatment‐related adverse events. Procedural information, including affected facial region, use of ultrasound or color Doppler guidance for procedural localization, target artery for intra‐arterial hyaluronidase injection, and the initial intra‐arterial hyaluronidase dose, was recorded when available. Ultrasound or color Doppler was not used as a standardized research comparator, and no quantitative ultrasound parameters were analyzed. Imaging‐derived perfusion metrics were extracted from the SPY‐Q output.

The HA‐based nature of the filler was confirmed through retrospective review of referral records, patient‐provided injection documentation, medical records from the injecting institution, or documented treatment history indicating HA filler injection. Cases in which the vascular occlusion was not attributable to HA filler injection were excluded. Because emergency evaluation and institutional rescue treatment occurred shortly after presentation, the time from filler injection to initial rescue treatment was used as the main timing variable.

Prior hyaluronidase treatment before presentation was recorded when documented in referral notes, patient history, or outside medical records, including the route when available. Because all patients were referred after receiving filler injections at other institutions, detailed information on filler brand, injection technique, injection volume, and the exact dose of prior hyaluronidase treatment was incompletely documented and was not included in the quantitative analysis.

### 2.5 Clinical Outcomes

The primary clinical outcome for treatment‐response assessment was treatment escalation after initial rescue treatment. Treatment escalation was defined as any additional active intervention after the initial rescue treatment for persistent local hypoperfusion or progressive ischemic tissue injury. For each patient requiring escalation, the specific intervention, timing of escalation, clinical rationale, and route of repeat hyaluronidase administration, if used, were recorded. Supportive observation alone was not classified as treatment escalation.

Final clinical outcome was recorded according to the last available clinical documentation and categorized as healed without visible sequelae, healed with scarring, or healed with hyperpigmentation. Safety was assessed by reviewing procedure‐related complications, indocyanine green‐related reactions, and other treatment‐related adverse events.

### 2.6 Statistical Analysis

Clinical continuous variables were summarized as mean ± standard deviation (SD) with minimum–maximum values when available. ICGA‐derived perfusion parameters were summarized as median and interquartile range (IQR), with minimum–maximum ranges additionally reported in the Results to preserve table readability. Categorical variables were summarized as counts and percentages. The primary analysis focused on paired changes in ICGA‐derived perfusion parameters before and after initial rescue treatment. The Wilcoxon signed‐rank test was used to compare ingress rate, TPP, and curve ingress between T0 and T1. All *p* values were two‐sided. Statistical analyses were performed using R software, version 4.4.3 (R Foundation for Statistical Computing, Vienna, Austria). A two‐sided *p* < 0.05 was considered statistically significant.

Curve ingress percent change was used to characterize the relative magnitude of early post‐treatment perfusion recovery. Ranked individual curve ingress percent changes were plotted to illustrate the distribution of tissue‐level perfusion changes across patients. Because treatment escalation occurred in only two patients, outcome‐related analyses were exploratory. These two cases were descriptively compared with the overall cohort distribution to evaluate whether their dynamic ICGA changes were located at the lower end of the observed response range. Because no standardized comparator modality, clinical severity score, capillary refill grading, or quantitative ultrasound/color Doppler parameters were available in this retrospective cohort, ICGA was not evaluated as a standalone diagnostic accuracy test. Therefore, no diagnostic accuracy analysis, formal comparator correlation, multivariable modeling, ROC analysis, or threshold derivation was performed.

## 3 Results

### 3.1 Patient Characteristics, Procedural Details, and Clinical Outcomes

A total of 26 patients with HA filler‐induced facial vascular occlusion were included in the final analysis (Figure 1). Patient characteristics, procedural details, and treatment escalation are summarized in Table 1. The cohort comprised 21 female patients (80.8%) and 5 male patients (19.2%). The mean age was 35.15 ± 6.59 years, with a range of 24–52 years. The mean BMI was 22.60 ± 3.82 kg/m^2^, with a range of 16.85–28.91 kg/m^2^.

**TABLE 1:** Patient characteristics and treatment escalation.

Variable	Value
Total patients	26
Sex	F: 21 (80.8%); M: 5 (19.2%)
Age, years	35.15 ± 6.59 (24–52)
BMI, kg/m^2^	22.60 ± 3.82 (16.85–28.91)
Time from filler injection to initial rescue treatment, days	1.81 ± 1.17 (1–6)
Treatment escalation after initial rescue treatment	2 (7.7%)

*Note:* Continuous clinical variables are presented as mean ± standard deviation (minimum–maximum), and categorical variables are presented as *n* (%). Treatment escalation was defined as any additional active intervention after initial rescue treatment for persistent local hypoperfusion or progressive ischemic tissue injury. BMI, body mass index.

The mean time from filler injection to initial rescue treatment was 1.81 ± 1.17 days, with a range of 1–6 days. Prior hyaluronidase treatment before presentation was documented in 17 patients (65.4%). When documented, prior treatment consisted of local hyaluronidase injection at the referring institution. Despite prior treatment, these patients continued to have persistent clinical signs of vascular compromise at presentation. Baseline ICGA demonstrated residual tissue‐level perfusion deficits within the affected cutaneous territory before the institutional rescue procedure.

All patients received a uniform initial intra‐arterial hyaluronidase dose of 1500 IU. Post‐treatment ICGA was generally performed approximately 15 min after completion of the initial intra‐arterial hyaluronidase injection according to the standardized peri‐treatment workflow. Treatment escalation after initial rescue treatment was required in two patients (7.7%). Both patients underwent escalation 48 h after initial rescue treatment because of persistent local hypoperfusion and progressive ischemic tissue injury. One patient underwent repeat intra‐arterial hyaluronidase injection followed by wound debridement, and the other underwent repeat intra‐arterial hyaluronidase injection.

Final clinical outcomes were classified as healed without visible sequelae in 22 patients (84.6%), healed with scarring in one patient (3.8%), and healed with hyperpigmentation in three patients (11.5%). The affected facial regions included the nasolabial folds in 13 patients (50.0%), frontal region in six patients (23.1%), nose in three patients (11.5%), upper lip in two patients (7.7%), and glabella in two patients (7.7%). The target artery was the facial artery in 18 patients (69.2%) and the supratrochlear artery in eight patients (30.8%). Patient‐level data, including affected facial region, target artery, prior hyaluronidase exposure, intra‐arterial hyaluronidase dose, treatment escalation, timing of escalation, and final clinical outcome, are provided in Table S1.

### 3.2 Representative Serial ICGA and Clinical Findings

**FIGURE 2:**
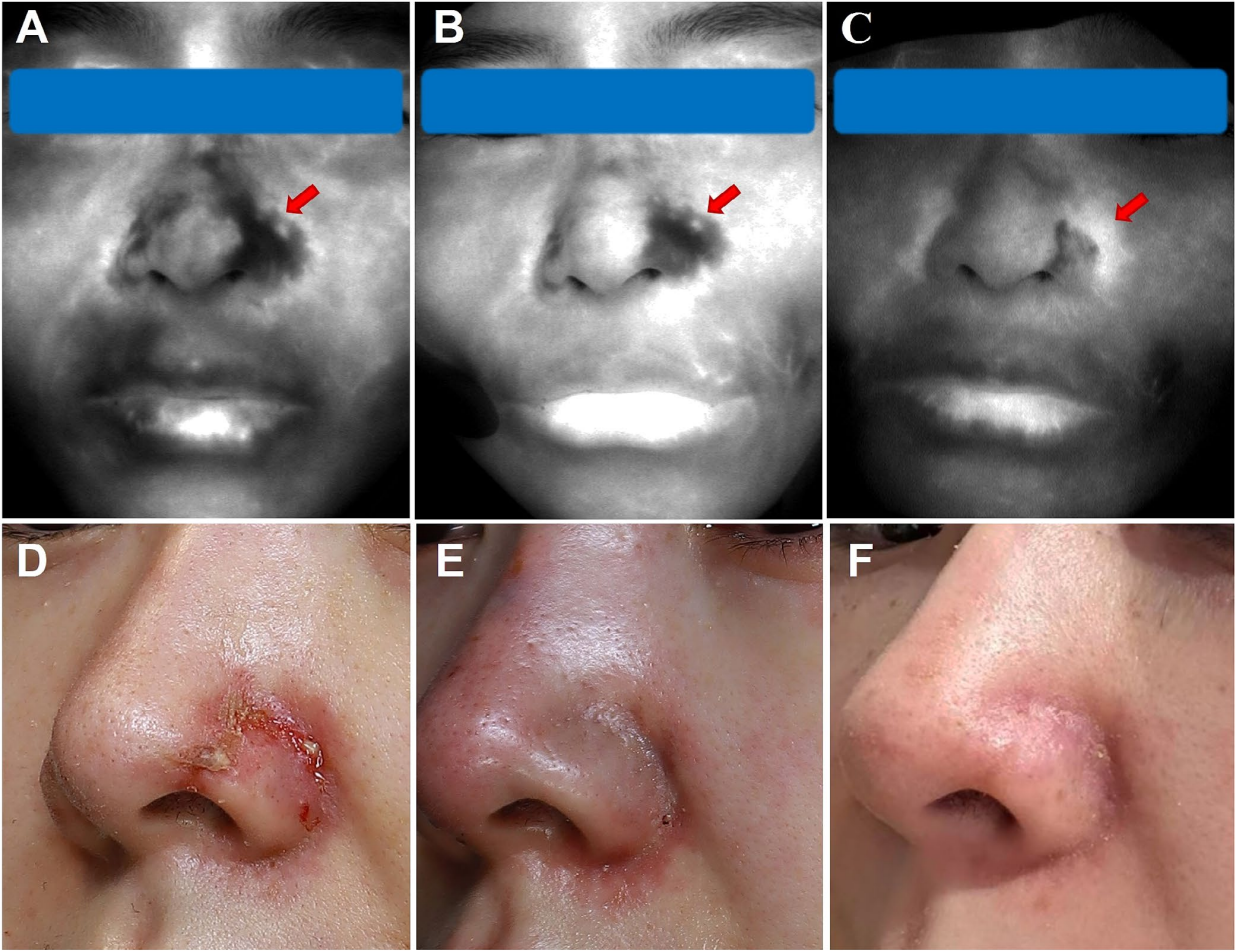
Representative serial ICGA and clinical images. Representative ICGA and clinical images in a patient with nasal/perinasal vascular occlusion after hyaluronic acid injection. (A–C) SPY/ICGA images at the time‐to‐peak perfusion phase: Baseline before treatment (A) approximately 15 min after completion of the initial intra‐arterial hyaluronidase rescue treatment (B) and 3 days after facial artery–guided intra‐arterial hyaluronidase treatment (C). Red arrows indicate the perfusion abnormality. (D–F) Corresponding clinical photographs before treatment (D), 7 days after treatment (E) and 1 month after treatment (F) ICGA, indocyanine green angiography.

Representative serial ICGA and clinical images are shown in Figure 2. Baseline ICGA demonstrated focal perfusion compromise within the clinically affected cutaneous territory. After initial rescue treatment, repeat ICGA showed increased fluorescence perfusion, indicating improved downstream cutaneous blood flow. Serial clinical photographs documented the corresponding evolution of ischemic skin changes during follow‐up. These images illustrate how ICGA visualized tissue‐level perfusion changes before and after rescue treatment.

### 3.3 Immediate Changes in ICGA‐Derived Perfusion Parameters

**FIGURE 3:**
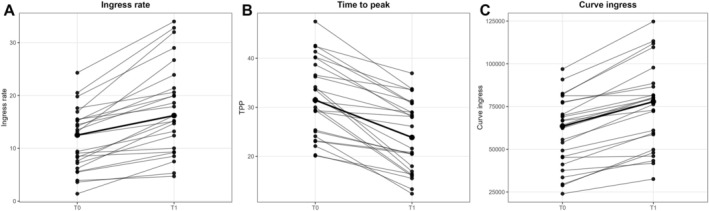
Paired changes in ICGA‐derived perfusion parameters. Paired changes in ingress rate (A; units/s), time to peak (B; seconds), and curve ingress (C; units·s) from baseline ICGA (T0) to post‐treatment ICGA (T1). T0 ICGA was obtained before initiation of intra‐arterial hyaluronidase injection. T1 ICGA was generally obtained approximately 15 min after completion of the initial intra‐arterial hyaluronidase injection. Thin lines represent individual patients, and thick black lines represent median values. ICGA, indocyanine green angiography; TPP, time to peak.

Paired comparisons of ICGA‐derived perfusion parameters before and after initial rescue treatment are summarized in Table 2 and Figure 3. After initial rescue treatment, paired ICGA assessment showed consistent improvement in tissue‐level perfusion. The median ingress rate increased from 12.50 (IQR, 7.30–15.35) at T0 to 16.20 (IQR, 10.60–21.20) at T1, with a median change of 4.85 (IQR, 3.00–8.57; *p* < 0.001). The median TPP shortened from 31.46 s (IQR, 25.14–38.14) to 23.87 s (IQR, 17.20–30.35), with a median change of −6.09 s (IQR, −11.69 to −3.93; *p* < 0.001). The median curve ingress increased from 63515.95 (IQR, 45364.60–75534.45) to 77862.55 (IQR, 58892.07–85363.50), with a median change of 14432.45 (IQR, 9891.28–20269.65; *p* < 0.001).

**TABLE 2:** Paired ICGA‐derived perfusion parameters before and after initial rescue treatment.

Parameter	Baseline ICGA (T0)	Post‐treatment ICGA (T1)	Change (T1−T0)	*p*
**Ingress rate, units/s**	12.50 (7.30, 15.35)	16.20 (10.60, 21.20)	4.85 (3.00, 8.57)	< 0.001
**TPP, s**	31.46 (25.14, 38.14)	23.87 (17.20, 30.35)	−6.09 (−11.69, −3.93)	< 0.001
**Curve ingress, units·s**	63515.95 (45364.60, 75534.45)	77862.55 (58892.07, 85363.50)	14432.45 (9891.28, 20269.65)	< 0.001

*Note:* Values are presented as median (interquartile range). Change was calculated as T1−T0 and is presented in the same units as the corresponding parameter. Curve ingress percent change was calculated as [(curve ingress at T1 − curve ingress at T0)/curve ingress at T0] × 100%. *p* values were calculated using the Wilcoxon signed‐rank test. ICGA, indocyanine green angiography; T0, baseline assessment; T1, post‐treatment assessment; TPP, time to peak.

The direction of change was concordant across all three ICGA‐derived parameters, indicating faster inflow, shorter perfusion delay, and increased cumulative fluorescence signal after initial rescue treatment. The ranges for ingress rate were 1.40–24.30 units/s at T0, 4.70–34.00 units/s at T1, and 0.80–15.10 units/s for paired change; corresponding ranges were 20.13–47.47, 12.40–36.93, and −16.67 to −1.20 s for TPP, and 23984.60–96911.90, 32587.90–124752.30, and 114.70–29823.30 units·s for curve ingress. Curve ingress percent change ranged from 0.14% to 72.25%.

### 3.4 Curve Ingress Percent Change and Treatment Escalation

**FIGURE 4:**
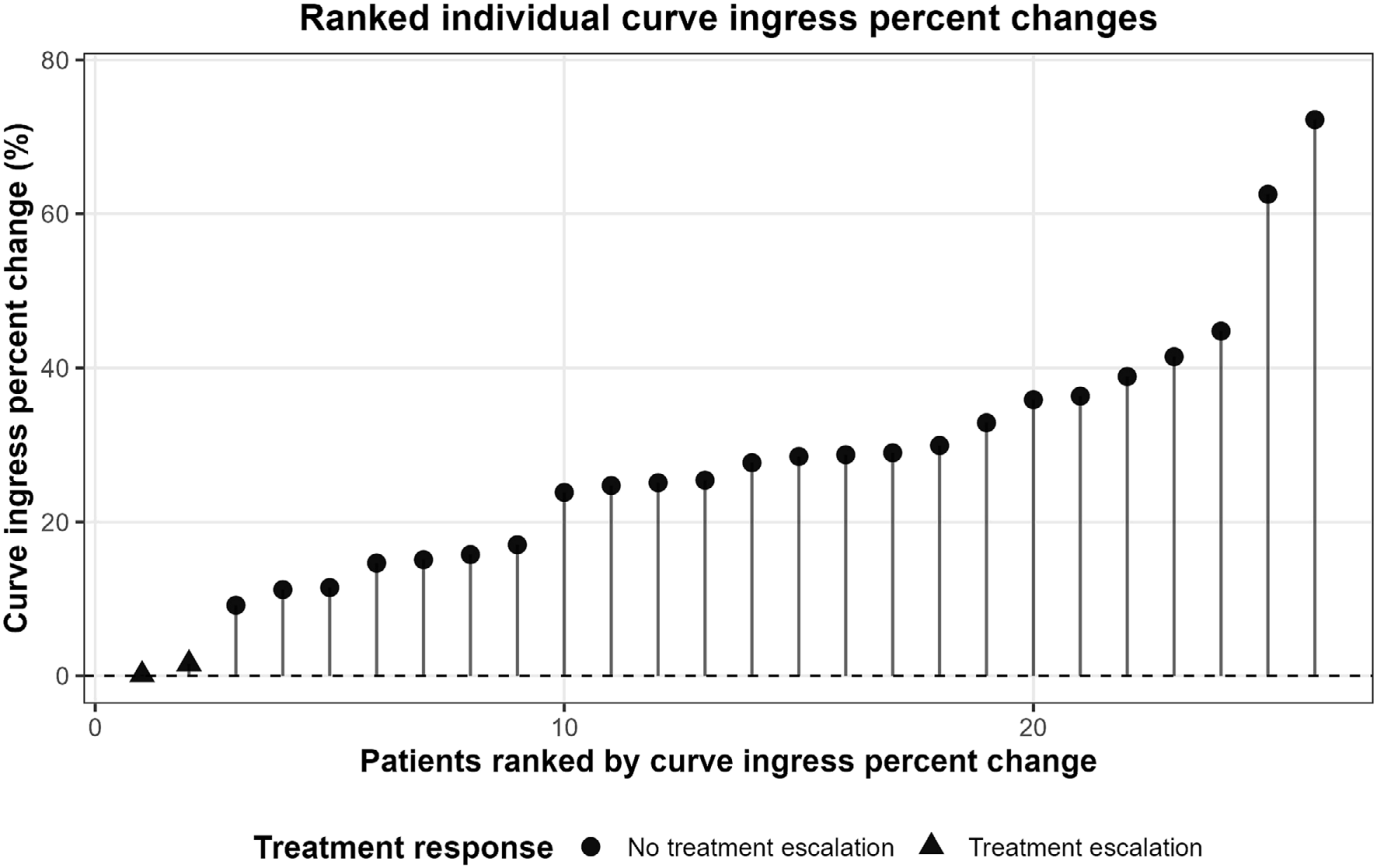
Ranked individual curve ingress percent changes. Curve ingress percent changes were calculated as [(curve ingress at T1 − curve ingress at T0)/curve ingress at T0] × 100% and ranked from lowest to highest after initial rescue treatment. Circles indicate patients without treatment escalation, and triangles indicate patients requiring treatment escalation. The dashed line indicates 0% change.

The median curve ingress percent change was 26.55% (IQR, 15.24%–35.12%), with individual values ranging from 0.14% to 72.25%. Ranked individual curve ingress percent changes are shown in Figure 4. Most patients demonstrated improvement in curve ingress after initial rescue treatment.

The two patients requiring treatment escalation had the lowest curve ingress percent changes in the cohort, at 0.14% and 1.57%, respectively. In both patients, minimal early improvement in curve ingress was accompanied by clinical evidence of persistent local hypoperfusion after initial rescue treatment.

### 3.5 Safety

No treatment‐related adverse events, including procedure‐related complications or indocyanine green‐related reactions, were documented during the intervention or within the available follow‐up period.

## 4 Discussion

In this exploratory single‐center retrospective feasibility study, ICGA demonstrated paired tissue‐level perfusion changes after initial rescue treatment for HA filler‐induced facial vascular occlusion. Three principal findings emerged. First, paired ICGA‐derived parameters changed consistently after treatment, with increased ingress rate, shortened TPP, and increased curve ingress, indicating improved downstream cutaneous perfusion. Second, curve ingress percent change provided a clinically intuitive measure of relative early reperfusion. Third, the two patients requiring treatment escalation showed the lowest curve ingress percent changes in the cohort, and this limited early improvement corresponded clinically to persistent local hypoperfusion after initial rescue treatment.

The main value of ICGA in this setting is its capacity to visualize and quantify downstream cutaneous perfusion. Acute vascular compromise after facial aesthetic injection may progress rapidly and can lead to substantial tissue injury if recognition or rescue is delayed [18]. Clinical evaluation remains essential for diagnosis and immediate management, but findings such as pain, blanching, livedoid discoloration, delayed capillary refill, and evolving ischemic change are partly subjective and may fluctuate during the early post‐treatment period. ICGA complements bedside assessment by visualizing tissue‐level perfusion and quantifying early hemodynamic change [19]. In the present cohort, the concordant increase in ingress rate, decrease in TPP, and increase in curve ingress support the interpretation of an overall reperfusion response rather than an isolated parameter fluctuation.

These findings are consistent with the broader use of ICGA in perfusion‐oriented surgical practice. ICGA has been applied for perforator localization, flap design optimization, intraoperative perfusion assessment, and tissue‐viability evaluation [20, 21, 22, 23]. Quantitative perfusion tools, including SPY‐based analysis, have also been used to evaluate perfusion intensity and perfusion territories in vascular and reconstructive settings [24]. In head and neck reconstruction, ICGA has been used to assess flap perfusion and support intraoperative decision‐making [25]. Although filler‐induced facial vascular occlusion differs from flap surgery, both scenarios require objective assessment of superficial tissue perfusion. The present study extends this principle to the early management of filler‐related facial vascular compromise.

The distinction between ICGA and ultrasound or color Doppler imaging is clinically important. Ultrasound and color Doppler imaging are valuable for identifying vascular anatomy, assessing flow when feasible, and guiding targeted hyaluronidase injection.6,7 However, ultrasound‐based assessment is partly operator‐dependent and may vary according to the examiner's experience, scanning technique, and interpretation of vascular findings. When performed using a standardized acquisition workflow, SPY‐based ICGA can provide visual and quantitative information on downstream cutaneous perfusion within the affected tissue territory. Successful arterial localization or targeted hyaluronidase delivery does not necessarily confirm adequate reperfusion of the skin. Therefore, ICGA may provide complementary post‐treatment information when clinicians need to determine whether tissue reperfusion is sufficient or whether closer surveillance, repeat perfusion assessment, or treatment escalation is warranted.

The intra‐arterial hyaluronidase protocol in this study should be interpreted within the specific clinical context of our referred patient population. Most patients were referred after initial management at outside institutions, and prior local hyaluronidase treatment before presentation was documented in 17 patients (65.4%). Despite prior local treatment, these patients continued to show persistent ischemic symptoms and clinical signs of vascular compromise, and baseline ICGA demonstrated residual tissue‐level perfusion deficits within the affected cutaneous territory.

In this clinical context, further repeated local hyaluronidase injections into already compromised tissue may increase local tissue tension and potentially aggravate secondary tissue injury. Therefore, intra‐arterial hyaluronidase was used as a selected institutional rescue strategy, based on our previously reported clinical experience, to deliver hyaluronidase toward the suspected vascular territory. Paired ICGA demonstrated improved fluorescence perfusion within the affected cutaneous territory after initial rescue treatment, supporting the feasibility of ICGA for monitoring tissue‐level reperfusion in this setting. Conventional early hyaluronidase‐based rescue remains central to management, and larger comparative studies are needed to define the optimal route and protocol of hyaluronidase administration.

Importantly, ICGA should be regarded as an adjunctive monitoring tool rather than as a prerequisite for treatment. In suspected filler‐induced vascular occlusion, clinical recognition and immediate rescue treatment should not be delayed for imaging. In our workflow, SPY‐based ICGA was incorporated into the standardized peri‐treatment workflow. Therefore, ICGA was used to document tissue‐level perfusion before and after initial rescue treatment without interrupting or delaying the rescue procedure. Its potential role is most relevant when objective documentation of tissue‐level reperfusion may help clarify treatment response, particularly when clinical signs remain equivocal, the extent of cutaneous ischemia is difficult to judge, or early reperfusion appears incomplete.

The two patients requiring treatment escalation showed minimal curve ingress improvement immediately after initial rescue treatment, suggesting persistent downstream hypoperfusion despite intervention. Both also had longer injection‐to‐treatment intervals than the overall cohort, with delays of 4 and 6 days compared with a cohort median of 1.50 days. Prolonged vascular compromise may contribute to downstream microvascular injury, including endothelial injury, local inflammation, tissue edema, impaired microcirculatory flow, or secondary thrombus formation around the HA embolic material. These changes may limit complete reperfusion after hyaluronidase‐based rescue alone. Because no histopathologic, angiographic, or standardized Doppler evidence was available, this explanation remains speculative. Nevertheless, limited early curve ingress improvement may indicate the need for closer monitoring and timely reassessment.

Curve ingress percent change may be useful because it normalizes the absolute change in curve ingress to baseline perfusion status and provides a simple measure of relative early reperfusion. Risk stratification for filler‐induced vascular occlusion remains challenging because clinical severity, anatomic territory, treatment delay, and rescue strategies may all influence tissue recovery [26]. Quantitative ICGA parameters have been used in other vascular settings to evaluate perfusion change and revascularization outcomes, supporting the broader concept that dynamic fluorescence‐derived parameters can reflect tissue‐level perfusion recovery [27]. In the present cohort, most patients demonstrated positive curve ingress improvement, whereas the two patients requiring treatment escalation were located at the lowest end of the ranked distribution. This separation supports the potential clinical relevance of dynamic ICGA changes, although larger prospective studies are needed before a predictive threshold can be defined.

The clinical significance of modest early curve ingress improvement should be interpreted in the context of concurrent clinical findings and follow‐up evolution. Several patients showed only modest positive curve ingress percent changes after initial rescue treatment but recovered without treatment escalation, indicating that early curve ingress percent change should not be used as a standalone threshold for clinical decision‐making. A modest positive change at the early T1 assessment may reflect the initial phase of ongoing reperfusion, particularly when clinical signs are stabilizing or improving. In contrast, the two patients requiring escalation showed near‐absent early improvement, with curve ingress percent changes of 0.14% and 1.57%, together with persistent clinical evidence of local hypoperfusion.

The timing of post‐treatment ICGA may also influence the magnitude of observed perfusion recovery. In this study, T1 ICGA was generally obtained approximately 15 min after completion of the initial intra‐arterial hyaluronidase injection to provide an early post‐treatment assessment without delaying clinical decision‐making. A more delayed T1 assessment, or serial ICGA assessment at multiple post‐treatment time points, might have demonstrated greater subsequent perfusion improvement in some patients, especially those with modest early improvement. Therefore, our findings should not be interpreted as defining the optimal timing of post‐treatment ICGA. Instead, they support further evaluation of early ICGA as an adjunctive tool for treatment‐response assessment and closer surveillance. Future prospective studies should evaluate whether delayed or serial ICGA time points improve prediction of tissue outcomes and treatment‐escalation decisions.

Other vascular imaging modalities may have roles in selected severe presentations. Superselective intra‐arterial thrombolysis and angiographic assessment have been described in severe injection‐related vascular events, particularly in vision‐threatening cases or suspected ophthalmic artery involvement [28, 29]. DSA can provide direct vessel‐level visualization and support endovascular intervention in selected patients [30]. More broadly, vascular imaging strategies continue to evolve, including attention to contrast‐related safety and noninvasive dynamic vascular imaging approaches [31, 32]. Compared with these modalities, ICGA is particularly suited for repeated peri‐procedural assessment of superficial cutaneous perfusion. Future workflows may integrate clinical assessment, ultrasound‐guided or angiography‐guided treatment when appropriate, and ICGA‐based tissue perfusion monitoring.

No treatment‐related adverse events, including procedure‐related complications or indocyanine green‐related reactions, were documented in this cohort. This provides preliminary reassurance regarding the procedural safety of ICGA in this setting. However, the study was not powered to evaluate uncommon adverse events, and ICGA should still be used according to standard contraindications and institutional safety protocols.

Several limitations should be acknowledged. First, this was a retrospective single‐center study, which may introduce selection bias and limit generalizability. Second, treatment escalation occurred in only two patients; therefore, the present study cannot establish a definitive predictive threshold for curve ingress percent change. Third, ultrasound and color Doppler imaging were used for vascular localization and procedural guidance rather than as standardized comparator tests. Quantitative ultrasound parameters were not prespecified or consistently recorded, precluding formal assessment of concordance between ultrasound findings and ICGA‐derived perfusion parameters. Fourth, treatment decisions were made in routine clinical practice and may have reflected both disease severity and clinician judgment. Fifth, detailed filler product information, injection technique, and vascular‐territory documentation were incomplete because all patients were referred after outside injections; therefore, the influence of these factors on ICGA‐derived parameters could not be evaluated. Finally, although quantitative parameters were extracted using SPY‐Q software, region‐of‐interest selection and image‐acquisition conditions may influence measurement consistency.

Despite these limitations, this study supports the feasibility of using ICGA to document early tissue‐level perfusion changes after rescue treatment for HA filler‐induced facial vascular occlusion. The two patients requiring treatment escalation showed minimal early curve ingress improvement, suggesting that dynamic ICGA parameters may provide adjunctive information for treatment‐response assessment. These findings should be interpreted as exploratory and require validation in larger prospective studies with standardized clinical and imaging comparators.

## 5 Conclusions

ICGA demonstrated paired tissue‐level perfusion changes after initial rescue treatment for HA filler‐induced facial vascular occlusion. The observed increases in ingress rate and curve ingress, together with shortened TPP, support its potential role as an adjunctive early perfusion‐monitoring tool. Limited early curve ingress improvement was observed in patients requiring treatment escalation, suggesting that dynamic ICGA parameters may support closer surveillance and treatment‐response assessment when interpreted with clinical findings. Larger prospective studies are needed to validate its role in clinical decision‐making.

## Author Contributions

Guangdi Li: conceptualization, investigation, data curation, methodology, writing – original draft; Guiwen Zhou: conceptualization, investigation, data curation, methodology, writing – original draft; Miao Wang: investigation, data curation, visualization, writing – review and editing; Hongfan Ding: investigation, data curation, visualization, writing – review and editing; Chongli Yu: formal analysis, methodology, visualization, writing – original draft, writing – review and editing; Qiang Fu: methodology, resources, supervision, writing – review and editing; Minliang Chen: conceptualization, supervision, project administration, resources, writing – review and editing. All authors reviewed and approved the final version of the manuscript and agree to be accountable for all aspects of the work.

## Funding

The authors have nothing to report.

## Disclosure

The authors have nothing to report.

## Ethics Statement

The authors confirm that the ethical policies of the journal, as noted on the journal's author guidelines page, have been adhered to and the appropriate ethical review committee approval has been received. The US National Research Council's guidelines for the Care and Use of Laboratory Animals were followed. Written consent was obtained for publication of identifiable clinical images.

## Conflicts of Interest

The authors declare no conflicts of interest.

## Supporting information


**Table S1:** Patient‐level clinical, procedural, treatment escalation, and final outcome details.
BMI, body mass index; HA, hyaluronic acid; HAse, hyaluronidase; ICGA, indocyanine green angiography. Time from filler injection to initial rescue treatment was reported because patients underwent emergency evaluation and rescue treatment shortly after presentation in the institutional workflow. All patients received a uniform initial intra‐arterial hyaluronidase dose of 1500 IU. Treatment escalation was defined as any additional active intervention after initial rescue treatment for persistent local hypoperfusion or progressive ischemic tissue injury. Final clinical outcome was recorded according to the last available clinical documentation.

## Data Availability

The data that support the findings of this study are available on request from the corresponding author. The data are not publicly available due to privacy or ethical restrictions.
